# New Drugs, Appliances, and Things Medical

**Published:** 1891-03-14

**Authors:** 


### NEW DRUGS, APPLIANCES, AND THINGS MEDICAL.

[All preparations, appliances, novelties, etc., of which a notice is desired, should be sent for The Editor, to care of The Manager, 140, Strand, London, W.C.]

### SWINBORNE'S ISINGLASS.

Nurses, cooks, mothers, and children, and most invalids, are fond of isinglass—if it be good. It should be good, or it ought not to be used at all. Calfs' foot jelly, when properly made, is very nice to take, and though not highly nutritious in itself, is an aid both to appetite and nutrition. Substances which are called isinglass are made from a considerable variety of raw materials, and those who know most about the isinglass family are most suspicious of certain members of it. Messrs. J. W. Swinborne and Co. have sent us samples of their isinglass, and we have tested them in several practical ways. Swinborne's isinglass is undoubtedly of the highest quality. It is excellently manufactured, and when properly mixed with jellies, soups, blanc manges, and other articles both for ordinary and for infants' and invalids' use, it imparts a beautiful clearness and smoothness to their appearance, and adds greatly to their palatability. It is not necessary for us to recommend the use of isinglass, because the article is in very general use already. What we do recommend is that when a substance of such doubtful manufacture is employed, care should be taken to purchase only the best. Most persons who use Swinborne's isinglass declare it to be the best. Our experiments lead us to coincide in that opinion.

### THE SANITARY AND ANTISEPTIC RESPIRATOR.

(B. Dranfield and Co., Corporation Oak, Nottingham, and Maw, Son, and Thompson, London.)

The improved form of the above has been forwarded to us for inspection. As we believe that it was at our suggestion that certain of the improvements were made, we are glad to notice this useful article again. It is made in flesh-coloured silk and wool or a plainer form in black and pink wool. It has a metallic perforated shield, which can be removed for cleaning, and a space for medicated cotton of any variety. The whole apparatus can easily be cleaned, and thus the chief objection to respirators is done away with. The cost, even of the most elaborate form, is low, so as to come within the reach of all classes The shape is sightly, and the pink-coloured one is hardly visible, thus doing away with the objection that some have for respirators.

### "ANGEL-WHITE" TOILET POWDER.

Mr. G. C. Blackwell, Liverpool, has forwarded to us for notice a sample of the above. It is a delicately-scented fine powder, free from gritty particles, metallic impurities, and irritating substances generally, and is well adapted for all purposes for which it is intended. More especially it is suited for all with delicate skins; ladies and infants will find it particularly useful and pleasant.

